# A Scoring Function for Monolayer-Protected Gold Nanoparticles Capable of Recognizing Small Organic Molecules in Solution

**DOI:** 10.1021/acs.jctc.5c01278

**Published:** 2025-10-24

**Authors:** Joseph Wallace, Laura Riccardi, Fabrizio Mancin, Marco De Vivo

**Affiliations:** † Molecular Modeling and Drug Discovery, 121451 Istituto Italiano di Tecnologia, via Morego 30, 16163 Genova, Italy; ‡ Department of Chemical Science, University of Padova, Via Marzolo 1, 35131 Padova, Italy

## Abstract

Ligand-coated gold nanoparticles (AuNPs) can act as self-organized nanoreceptors capable of selectively recognizing small organic molecules (analytes) in solution. This ability can be applied in several fields, with NMR chemosensing being a notable example. To advance the rational design of such AuNP-based nanosensors, we present a data-driven scoring function to rapidly estimate AuNP–analyte binding affinities, thus allowing fast *in silico* prescreening of ligand-coated AuNP sensors. This scoring function implements chemical similarity, hydrophobicity, and charge complementarity as key molecular descriptors, demonstrating excellent predictive accuracy when validated against experimental data (*R* ^2^ = 0.85, MAE = 0.45 kcal/mol). Enhanced sampling molecular dynamics on representative systems revealed that ligand flexibility, monolayer packing, and hydrogen bonding critically shape binding interactions, particularly for weak binding systems. Together, these data-driven and atomistic insights offer a robust framework for the rational design and optimization of AuNP-based nanosensors.

## Graphical abstract

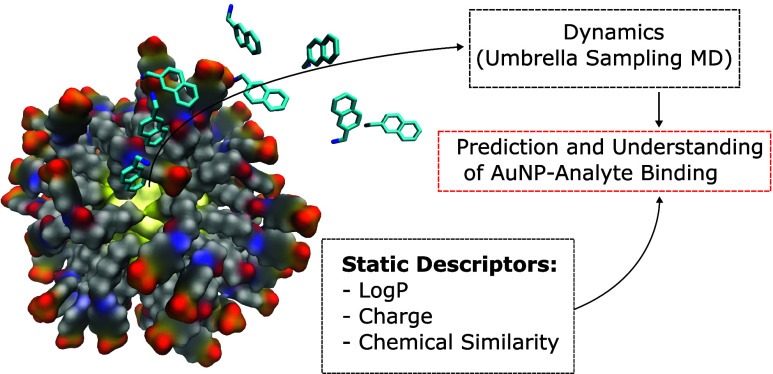

## Introduction

Chemosensing is the ability to noninvasively detect and quantify molecular species in complex environments with high specificity and sensitivity.^1^ Significant advancements have been made in this field, such as the development of fluorescent chemosensors.^2–7^ Over the past two decades, gold nanoparticles (AuNPs) have emerged as a versatile platform for chemosensing, paving the way for numerous innovative approaches.^8,9^ Many early examples relied on the surface plasmon resonance (SPR) properties of gold nanoparticles to result in aggregation-induced or growth-induced color changes.^10,11^ Recently, such optical properties of gold nanoparticles have also been utilized in lateral flow tests.^12–14^ Other examples based on different nanoparticle properties include indicator displacement assays (IDA) based on the ability of AuNPs to quench the emission of dyes, nanozymes for colorimetric tests, and modulation of quantum dots’ emission.^15–17^

Traditional chemosensors suffer from selectivity issues, i.e., the inability of the sensor to indicate the exact identity of the analyte, as the sensors’ response is solely derived from the change in their properties upon analyte binding. AuNPs provided a solution to this by enabling protocols for nanoparticle-assisted NMR chemosensing.^18–22^ The major benefit of such protocols is the ability to probe the guest molecule directly, thereby reducing the possibility of false positives and, in several cases, eliminating the need for analytical standards.^23^

In this context, a key aspect of the nanoparticle-assisted chemosensing approach is the engineering of nanoparticles with molecular recognition abilities. Indeed, the key transfer of magnetization (or saturation) from the nanoparticle to the analyte, which allows the separation of the signals of the analytes from those of other molecules present in the sample, occurs within a nanoparticle-analyte host–guest complex. Molecular recognition abilities are usually endowed to AuNPs by tailored coating ligands. Upon formation of a self-assembled monolayer surrounding the gold core, such ligands can form transient binding pockets to host the guest analyte in solution.^23,24^ Designing self-assembled monolayers of ligands with the desired host properties on the surface of AuNPs is, however, not a trivial task. Monolayers of sensing-sized AuNPs (2–10 nm) are typically composed of 50–1000 ligands. Compared to protein systems, self-assembled monolayers are generally more dynamic and structurally less defined, resulting in a limited number of experimentally determined structures.^25–38^ Yet, approaches through rational design for ligand-coated AuNPs have been put forward, resulting in AuNPs with binding affinities in the low μM range toward small organic analytes.^39^ Strategies used evolved from simplified binding sites models,^20,39^ to molecular dynamics-assisted rational design^23,24,40^ and eventually to high-throughput computational screenings.^41^ With this last method, we have recently designed and realized a novel tripeptide-based coating monolayer with the ability to detect the neuroblastoma biomarker catecholamine 3-methoxytyramine (3-MT) at concentrations below 25 μM.^41^

In the search for even more effective methods for the fast and efficient design of AuNPs with tailored molecular recognition abilities, we present here a rapid and interpretable scoring function that predicts AuNP-analyte binding affinities on the basis of hydrophobicity, charge complementarity, and chemical similarity. Additionally, enhanced sampling molecular dynamics (MD) simulations provided insights into how monolayer dynamics and hydrogen bond networks of ligand-coated AuNPs influence binding events.

This work establishes a data-driven framework for rational AuNP design, enabling efficient screening and a deeper mechanistic understanding of AuNP-analyte interactions.

## Results and Discussion

### Understanding the Data Set through Chemical Clustering Analysis

Initially, we searched the literature for AuNP-analyte systems that met the following criteria:

1. Known experimental binding free energy (Δ*G*).
2. A gold core size between 1.4 and 2.0 nm, broadly corresponding to the Au_144_(SR)_60_ structure.

After this selection, we retrieved 32 AuNP-analyte systems, with experimental Δ*G* ranging from −2.85 to −8.71 kcal/mol. Each capping ligand, in its isolated form, was also characterized by its hydrophobicity expressed as log *P* (logarithm of the 2-octanol/water partition coefficient), as predicted computationally via RDKit through the Crippen Log *P* module.^42,43^

To understand the factors governing AuNP-analyte binding, we first applied a chemical clustering analysis based on Tanimoto similarity to all the AuNPs and analytes independently (see the Computational Materials and Methods section for details).

The nanoparticle ligands fell into three distinct clusters (Figure 1A): (1) The first cluster (orange) comprised ligands that feature an alkyl linker, a dipeptide segment, and a methyl ester-capped aspartic acid, rendering them negatively charged at neutral pH. Despite a wide range of hydrophobicity (log *P* from −2.07 to 1.61), these ligands exhibited intermediate binding free energies, clustering around −5 kcal/mol (Figure 2); (2) The second cluster (green) consisted mainly of ligands with alkyl linkers (except for one ether-containing ligand), a central amide/carbamide/carbamate group, and a polyether terminal group; these neutral and moderately hydrophobic ligands (log *P* between 0.31 and 1.89) consistently correlated with the lowest binding affinities observed in our data set (Figure 2); (3) The third cluster (red) included ligands that, while sharing an alkyl linker, differed in their terminal functionalities, either negatively charged sulfonate or positively charged ammonium. Notwithstanding the charge, these ligands were highly hydrophobic (log *P* = 1.82 to 2.59) and correlated with the highest binding affinities (Figure 2). It is worth noting that, within each cluster, the binding affinities remained consistently similar, underscoring how chemical similarity can encode this critical property for predictive modeling into a reduced-dimensional space.

**Figure 1:**
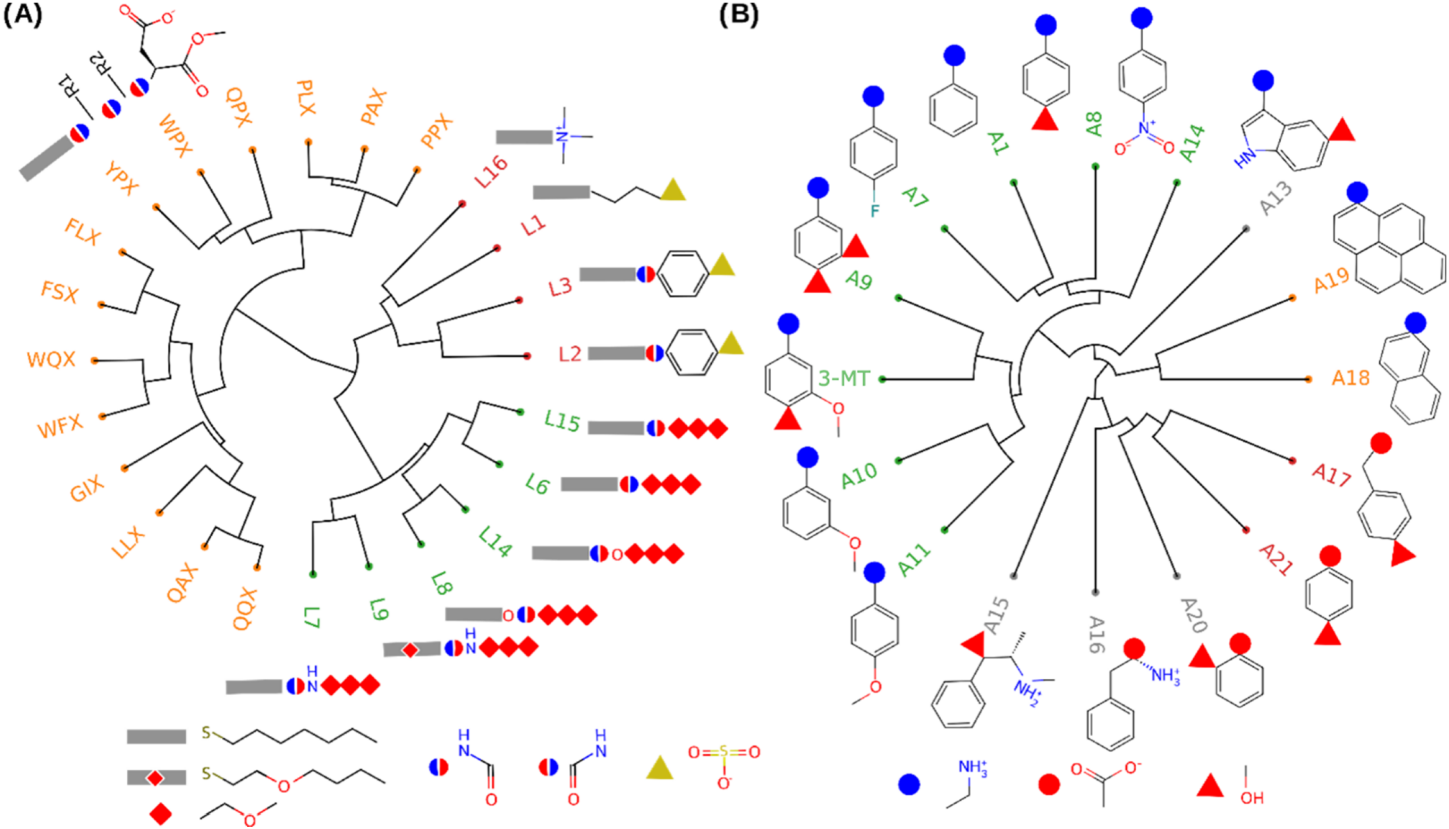
Molecular structures and their hierarchical Tanimoto similarity clustering, for all the ligands (A) and analytes (B). The amino acid-based ligands (orange cluster in (A)) share a common scaffold: an aliphatic linker, two amino acids (side chains R1 and R2, identified by the first two letters in the name), and an aspartate methyl ester capping (common terminal X in the name).

**Figure 2:**
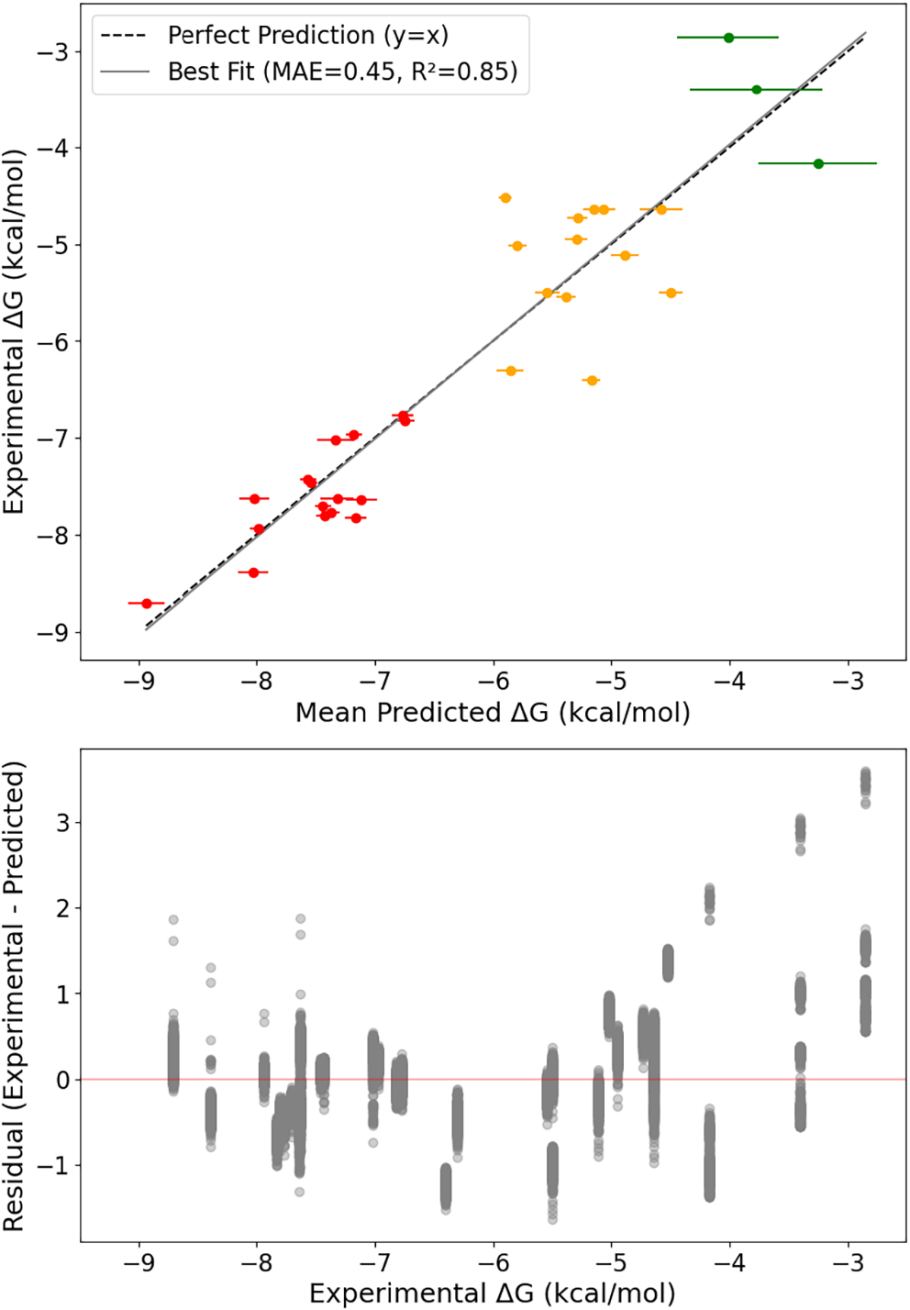
Ridge regression predictions of binding free energy (Δ*G*) vs experimental (Δ*G*) using repeated 5-fold cross-validation (1000 repeats) with inner CV for α selection. Top: Mean predicted vs experimental (Δ*G*). Dashed line = perfect fit (*y*=*x*). Solid line = best fit to the data (*R* ^2^ = 0.85). Points are colored according to the ligand clustering (Figure 1A). Cluster-wise metrics: Red cluster: MAE = 0.27, *R* ^2^ = 0.59, *n* = 16; yellow cluster: MAE = 0.55, *R* ^2^ = −0.32, *n* = 13, green cluster: MAE = 0.80, *R* ^2^ = −1.60, *n* = 13 (see Figure S1). Bottom: Residuals (experimental - predicted ΔG) plotted against experimental (Δ*G*).

Notably, the analytes used in this study were based on functionalized aromatic rings. Of the 16 total analytes studied, 1 contains a pyrene, 1 a naphthalene, 1 an indole, and 13 a phenyl ring. When looking at the chemical similarity across the analytes, one large cluster emerges (Figure 1B, green). This cluster is largely based on a shared benzylammonium scaffold, typically functionalized with an oxygen-containing functional group, although fluoro- and nitro groups were present in some analytes (A14 and A7). Second, two small clusters can be observed. One containing all fused polycyclic scaffolds (Figure 1B, orange), and the second sharing a hydroxybenzene-carboxylate motif (Figure 1B, red). The analyte A13, with its indoxyl ring, was clustered alone, as were analytes A15 and A16 with their extended side chains when compared to all other ligands (Figure 1B, gray). Finally, A20 is also clustered alone due to the differing position of its side chains in comparison to analytes A21 and A17. Together, both the ligand and analyte chemical clustering highlight how complex chemical structures can be encoded in a meaningful way through a single number: a chemical similarity score.

### A Scoring Function for AuNP-Analyte Binding

Based on this analysis, to efficiently predict the binding affinities of small molecules to ligand-protected AuNPs, we developed a scoring function based on three key molecular descriptors: hydrophobicity, charge complementarity, and chemical similarity. The scoring function was constructed using ridge regression, a regularized linear model that reduces the level of overfitting. Model hyperparameters were optimized using nested k-fold cross-validation, ensuring robust performance despite the relatively small data set. We emphasize that the scoring function was trained exclusively on experimentally characterized binders with measurable Δ*G* values; thus, it is not designed to classify or predict binders that fall below the experimental detection limit.

Each AuNP-analyte system was represented as a feature vector of size 5, composed of three molecular descriptors:

1. Hydrophobicity of the AuNP ligand and the analyte, quantified using log *P*, to account for hydrophobic contributions from analytes and ligands.
2. Charge complementarity, defined as the absolute difference between ligand and analyte charge, to account for electrostatic contributions.
3. Chemical similarity of the AuNP ligand and the analyte, encoded through Tanimoto similarity scores, to encode static structural relationships between different AuNP-analyte pairs.

The scoring function achieved excellent predictive accuracy when applied to a data set of 32 AuNP-analyte complexes with experimentally validated binding free energies (Δ*G*), either reported directly or derived from equilibrium constants using the standard thermodynamic relation (Δ*G* = −*RT* ln* K*). The resulting correlation between predicted and experimental Δ*G* values (*R* ^2^ = 0.85, MAE = 0.45 kcal/mol, Figures 2 and S1) demonstrates the ability of simple molecular descriptors to capture key trends in AuNP-analyte binding. These descriptors reflect long-recognized drivers of molecular recognition, particularly hydrophobic collapse and electrostatic attraction, which have proven especially relevant in ligand-coated AuNP-based systems.^20,23,44^

We report both scaled and unscaled regression coefficients to quantify the feature significance and enable the direct application of our scoring function (Table 1). Ridge regression employs an L_2_ penalty to shrink all coefficients toward zero, thus reducing variance and potential overfitting.^45,46^ Since all variance inflation factors are <3,^47^ the higher the value of the absolute scaled coefficient, the greater its impact on Δ*G*. This allows ranking features based on each contribution to the final prediction.^46–48^ Accordingly, charge difference ranks as the most critical feature used here, followed by ligand log *P*, analyte log *P*, ligand Tanimoto, and analyte Tanimoto (Table 1).

**Table 1:** Variance Inflation Factors, Scaled Coefficients, and Unscaled Coefficients for the Final Scoring Function

descriptor	variance inflation factors	scaled coefficient	unscaled coefficient
Intercept	-	-	0.5450
Charge Difference	1.83	–0.753	–2.5822
Ligand Log *P*	2.67	–0.581	–0.4023
Analyte Log *P*	1.34	–0.471	–0.4902
Ligand Tanimoto	2.56	–0.410	–4.7748
Analyte Tanimoto	2.05	0.044	0.6395

### Ligand Flexibility and Binding Dynamics Distinguish High- and Low-Affinity Complexes

While our scoring function accurately predicts binding affinities for most systems, its performance declines near the experimental limit of detection, i.e., in the case of weak binders. This suggests that static descriptors alone may be insufficient to capture the nuanced interactions that govern low-affinity binding. To directly investigate this regime, we selected six representative AuNP-analyte complexes for enhanced sampling MD: four with experimentally measured Δ*G* values and two with no detectable binding under experimental conditions (weak binders).

These six systems were chosen to isolate the effect of subtle, but impactful, structural differences: a single amide bond inversion between two otherwise identical ligands, L2 and L3 (Figure 1A). Each ligand was paired with the same three analytes (A10, A18, and A19), allowing direct comparison of L2- and L3-analyte interactions. Despite their near-identical composition, L2-capped AuNPs exhibited detectable binding to all three analytes, while L3 only showed binding toward a single analyte, A19, under experimental conditions.

To probe these effects directly, we applied our recent screening protocol based on steered molecular dynamics (sMD),^41^ extending it here with the addition of umbrella sampling molecular dynamics (US-MD) to recover semiquantitative potential mean forces (PMFs). We used the center-of-mass distance between AuNPs and analytes as a reaction coordinate (RC) to approximate the free energy landscape of analyte dissociation. However, while this approach performed well for strongly binding systems, as in earlier work, its predictive accuracy broke down for weakly bound complexes (Figures S2–S3). This discrepancy suggests that a simple RC may miss critical factors, particularly conformational penalties associated with ligand reorganization that are needed to form binding pockets. Indeed, we previously demonstrated that, in the case of low-affinity AuNP-analyte complexes, monolayer packing and dynamics can also significantly affect the affinity of guests, as densely packed ligand shells must pay higher conformational costs to form the binding pockets.^23,24,40^

Nonetheless, the MD trajectories remained highly informative. Dihedral angle analysis revealed that the amide inversion in L3 restricts the conformational flexibility. L2-capped AuNPs sampled four distinct phenyl-amide dihedral states, with the N–H bond adopting orientations between planar and perpendicular (Figure 3A, purple). Meanwhile, L3 systems were limited to two states, with the carbonyl oxygen remaining planar to the phenyl ring (Figure 3A, orange).

**Figure 3:**
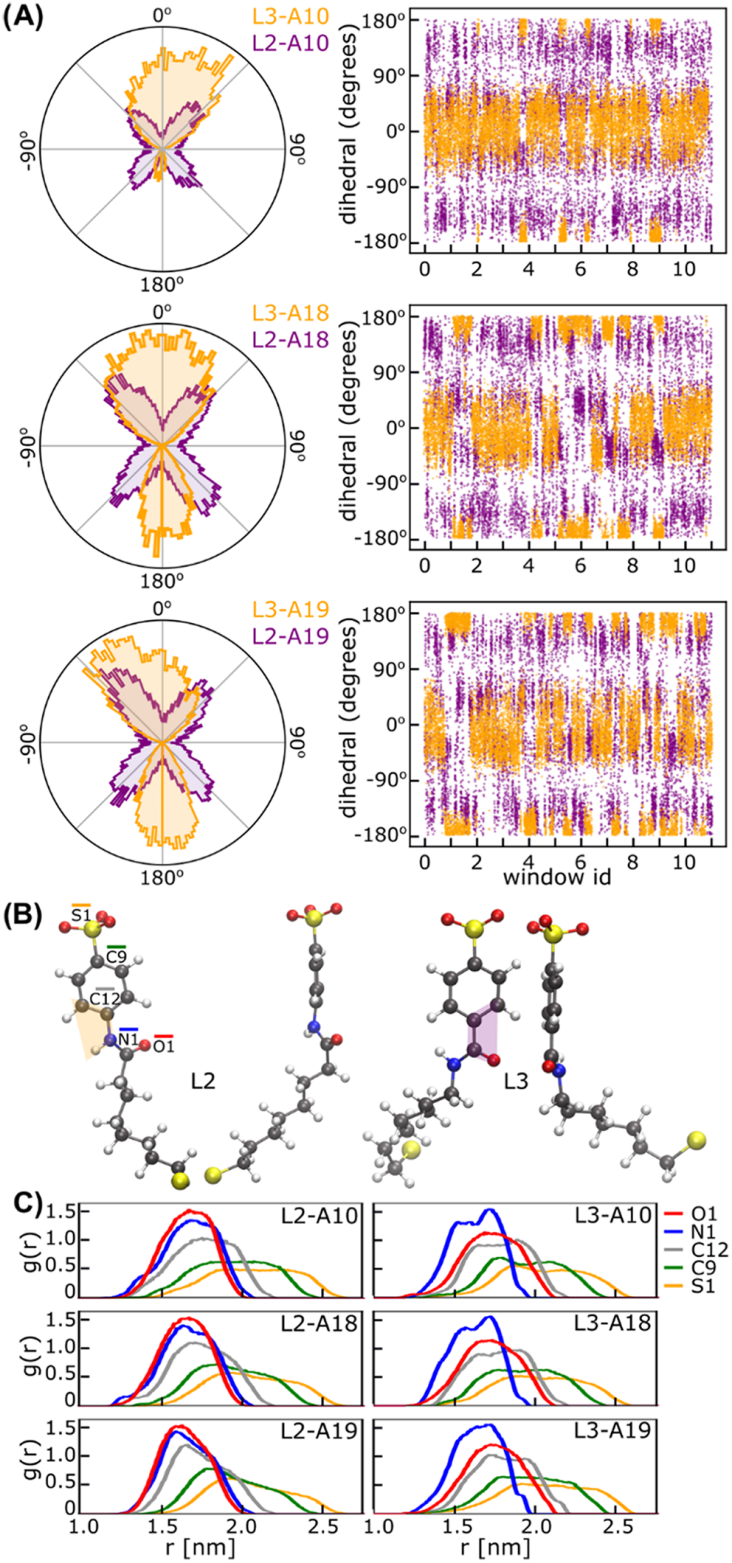
Amide-phenyl conformational dynamics and monolayer packing in L2 vs L3 AuNPs. (A) Conformational dynamics of the amide-phenyl dihedral angle in L2 vs L3 ligands. Right: Time series of the same dihedral angle for a representative ligand. (B) Representative low-energy conformers of each ligand. (C) Radial distribution functions (RDF) of terminal atoms within ligand monolayers.

Importantly, L2-capped nanoparticles exhibited a much higher transition frequency between states. This system also showed a greater root-mean-square deviation of all atomic positions when compared to L3-capped nanoparticles (Figures S4 and S5), indicating enhanced conformational flexibility. Consistent with this, atomwise RMSF analysis showed that, while both ligands are quite flexible across their chain with fluctuations increasing radially outward from the gold surface, the L2 capping moiety is more flexible than that of the L3 one (Figure S6). Radial distribution function (RDF) analysis (Figure 3C) further demonstrated that amide inversion affects monolayer packing. In L2, amine nitrogen (N1) and carbonyl oxygen (O1) have almost identical distribution, whereas in L3, the nitrogen is distributed deeper within the monolayer, suggesting a more extended ligand conformation. Such differences in ligand flexibility, invisible to static descriptors, likely underpin the experimentally observed divergence in binding behavior.

Furthermore, we analyzed the H-bond interactions between the analytes and the ligands, a parameter that has been shown to correlate well with binding affinity.^49^ We noted that systems with detectable binding (and greater ligand mobility) consistently exhibited more hydrogen bond interactions (Figure 4, green bars). In particular, the improved analyte affinity was coupled to a greater number of H-bonds with the sulfonate groups (Figure 4, orange bars). On the contrary, weakly binding systems (L3-A10, L3-A18) formed more hydrogen bonds with amide carbonyl groups (Figure 4, blue bars). This H-bonding pattern likely arose from the greater surface exposure of the L3 carbonyl group (Figure 3C). On the other hand, the reduced flexibility of L3 restricted the ability of analytes to form simultaneous interactions with the sulfonate headgroup and the hydrophobic interior of the monolayer, resulting in undetectable binding in L3-A10 and L3-A18. Notably, L3-A19 deviated from this pattern. The pyrene-containing molecule compensated for the reduction in monolayer mobility through more extended hydrophobic interactions, allowing detectable binding and restoring the hydrogen bonding network. In addition, we quantified interligand, i.e., within the monolayer, hydrogen bonds, which consistently showed higher H-bond counts for L2 compared to L3. This indicates that these transient contacts help maintain a cooperative and adaptable monolayer environment (Figure S7).

**Figure 4:**
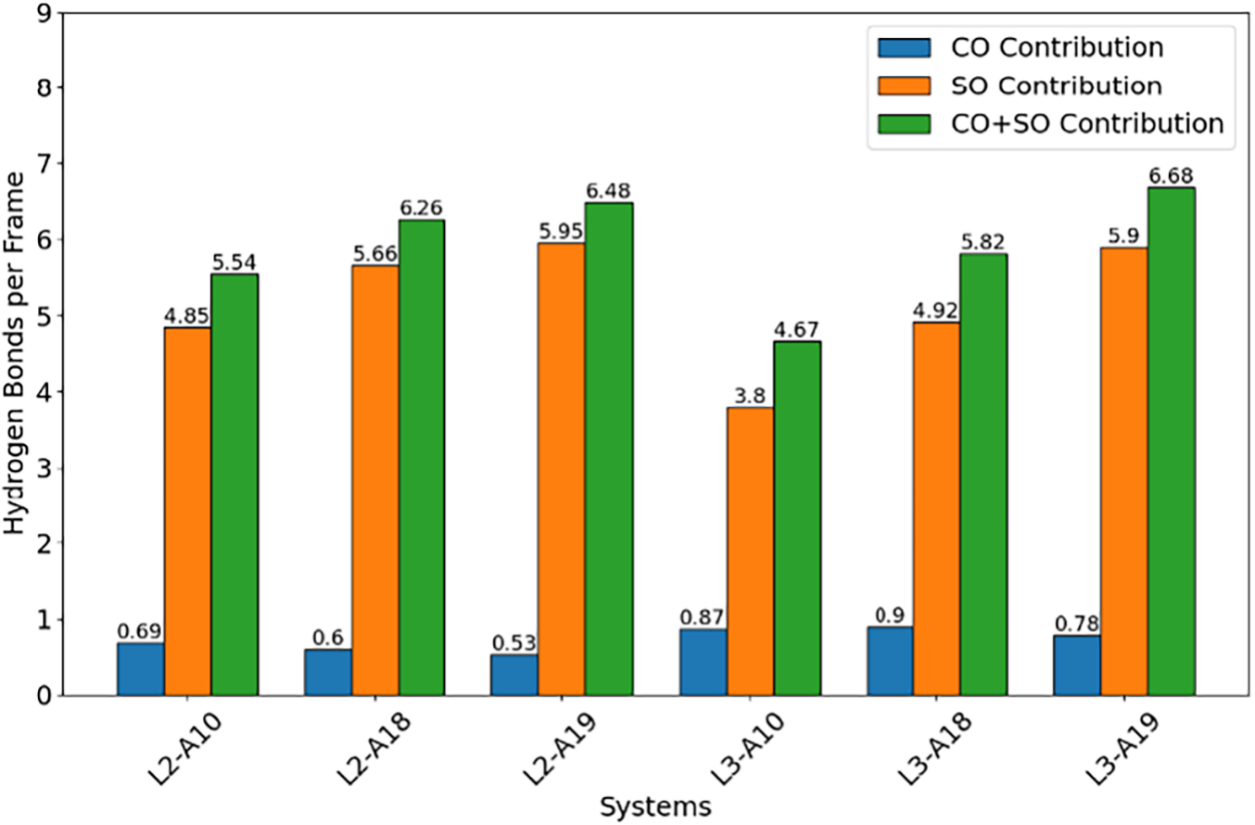
Average hydrogen bonds per frame for each of the 6 systems are broken down into those between analyte and carbonyl oxygen (CO contribution), the analyte and the sulfonate oxygens (SO contribution), and the combined (CO + SO contribution).

These findings highlight the crucial role of hydrogen bonding in modulating the binding affinity. Systems with dynamic and flexible ligand-analyte interfaces exhibited a greater binding affinity by stabilizing extensive hydrogen bonding interactions. Even subtle modifications, such as amide orientation, could significantly alter binding by restricting ligand flexibility and disrupting hydrogen bonding formation. These results underscore the need to integrate MD-derived insights into predictive models for atomic-level understanding of complementary point interactions that control host–guest affinity in ligand-protected AuNPs.

## Conclusions

In this study, we developed a data-driven scoring function for predicting AuNP-analyte binding affinities based on molecular descriptors, achieving high predictive accuracy (*R* ^2^ = 0.85 and MAE = 0.45 kcal/mol). This approach enabled the rapid prescreening of candidate nanoparticle systems, significantly reducing computational costs toward experimental testing.

However, while the model effectively ranked measurable binding affinities, it failed in estimating low affinities. The same result was obtained with sMD screenings. This suggests that both methods could underestimate the costs for the reorganization of the monolayer necessary for analyte binding. Static molecular descriptors do not allow an atomic-level comprehension of complementary guest–host–point interactions, but umbrella sampling molecular dynamics (US-MD) simulations revealed that ligand flexibility, hydrogen bonding, and monolayer dynamics are critical determinants for analyte binding. Notably, we found that small modifications of the ligand structure, such as the inversion of an amide orientation, can dramatically alter ligand dynamics, impacting analyte interactions and binding strength. Nonetheless, the approach remains constrained by the current limited data set size (32 systems). Predictive accuracy is more challenging for weak binders, where conformational penalties and transient interactions are more difficult to capture. We show that simple molecular descriptors capture the dominant binding trends while they fall short in resolving the subtleties of low-affinity systems. Addressing this will require expanded experimental data sets, e.g., for their use with machine learning methods. In summary, this work establishes a new framework for rational AuNP design, enabling efficient screening and a deeper mechanistic understanding of AuNP-analyte interactions.

## Computational Materials and Methods

### Chemical Similarity

Tanimoto similarity was assessed for the ligands and analytes as two separate groups. This enabled the visualization of structural relationships among ligands and analytes in a pairwise manner. For analytes, similarity was calculated relative to the phenol scaffold (SMILES string: “**Oc1ccccc1**”). For ligands, similarity was calculated relative to a heneicosanethiolate scaffold (SMILES string: “**CCCCCCCCCCCCCCCCCCCCC­[S-]**”**).** In both cases, SMILES strings were derived from GROMACS starting structure files using a custom Python script leveraging the MDAnalysis package. Each SMILES string was converted into an RDKit molecular object, to ensure canonical representation, followed by the generation of 1024-bit RDKit molecular fingerprints.^50^ Tanimoto coefficient, *T*, is then given by

1 $$T=\frac{c}{a+b-c}$$

where *a* is the number of bits set to 1 in the first molecule, *b* is the number of bits set to 1 in the second molecule, and *c* is the number of bits set to 1 in both molecules. To further analyze chemical similarity, hierarchical clustering was performed using SciPy’s dendrogram functionality, highlighting 3 main structural clusters (Figure 1).

### Ridge Regression

A total of 32 gold nanoparticle systems with associated experimental binding affinity values between nanoparticles and various small molecules were used as a data set. Each system had associated features, including log *P*, calculated using RDKit. Tanimoto similarity scores were computed for analyte molecules using RDKit fingerprints, with user-defined consensus molecules representing two ligand systems: a linear alkyl thiol (LSM) and an aromatic thiol (ASM). RDKit was utilized to generate molecular fingerprints and calculate Tanimoto similarity against these consensus molecules. Charge differences between nanoparticle ligands and analytes were calculated at pH 7.4 by using the Maestro Epik module. Log *P* values were computed using RDKit Crippen log *P* module.^42,43^ In order to estimate variance inflation factors for the model, the statsmodels package was utilized.

Ridge regression models were developed to predict binding affinities (Δ*G* values). The data set was split into five folds using k-fold cross-validation. Models were trained on four folds, with the fifth fold used for validation to assess model performance on unseen data. Repeated k-fold cross-validation was performed with 1000 iterations to evaluate the variability of predictions and to compute confidence intervals for metrics. Feature scaling was applied using standardization, ensuring a mean of 0 and unit variance across features.

Hyperparameter tuning was conducted using nested cross-validation, where the optimal regularization parameter (α) was determined via grid search across logarithmically spaced values ranging from 10^–6^ to 10^6^. For each split, ridge regression models were fitted using scikit-learn’s RidgeCV class, and predictions were collected. Model performance was evaluated by using mean squared error (MSE), mean absolute error (MAE), root-mean-square error (RMSE), and *R* ^2^. Results were visualized with scatter plots of predicted vs experimental Δ*G* values, including residual plots.

The final ridge regression model, after training, can be expressed in the following form:

$$\Delta G=0.5450+\left(-2.5822\cdot \mathrm{ChargeDifference}\right)+\left(-0.4023\cdot {\log P}_{\mathrm{Ligand}}\right)+\left(-0.4902\cdot {\log P}_{\mathrm{Analyte}}\right)+\left(-4.7748\cdot {\mathrm{Tanimoto}}_{\mathrm{Ligand}}\right)+(0.6395\cdot {\mathrm{Tanimoto}}_{\mathrm{Analyte}})$$

where Δ*G* is the free energy of binding expressed in kcal/mol, Log *P* is the Log *P* of either analyte or ligand, ChargeDifference is the difference in charge between a single ligand and a single analyte molecule, and Tanimoto is the chemical similarity score for either the ligand or analyte.

Custom Python code leveraging scikit-learn, pandas, matplotlib, and RDKit was employed for all analyses. Progress tracking during cross-validation was facilitated by tqdm, providing real-time updates.

### Molecular Modeling and System Setup

In addition to the 32 systems with experimental binding affinities used to create the scoring function, for molecular modeling and simulation, we included an additional 2 systems (L3–10 and L3-A18), which showed no detectable binding experimentally.

Ligand molecules were first generated in a fully extended conformation, with the most probable protonation state determined via Epik module,^51^ at neutral pH. Atomic partial charges were then derived from the restrained electrostatic potential (RESP) method,^52^ with bonded parameters from the General Amber Force Field (GAFF).^53^

The NanoModeler web server was used to generate initial conformations of ligand-protected gold nanoparticles (AuNPs).^54,55^ In this work, all nanoparticles were modeled as 2 nm Au_144_(SR)_60_ nanoparticles based on the structure from Lopez-Acevedo et al.^30^ Nanomodeler produced the required parameter files for the ligand-protected AuNPs needed for further simulation and is described elsewhere.^54^ Briefly, inner quasi-static gold atoms of the AuNPs were modeled as neutral spheres with Lennard-Jones parameters taken from Heinz et al.,^58^ and the staple-like motifs at the gold–sulfur interface were modeled with the AMBER-compatible parameters derived previously.^56^

All analyte structures were also generated utilizing Maestro Epik for protonation state determination at neutral pH,^51^ RESP for partial atomic charges^52^ and the GAFF force field.^53^

All systems then included a single ligand-protected AuNP with ten identical analyte copies. Nanoparticle and analyte files were then merged to create a single copy of all files required for simulation (structure and parameter files). Analytes were initially placed two-thirds of the way into the monolayer (or one-third of the monolayer length away from the gold surface) via in-house Python scripts. Orientations of analytes were determined randomly but with the use of a random seed, and a minimum distance between all atoms of 0.15 nm was maintained to avoid intermolecular clashes.

Each AuNP system was then placed in the center of a dodecahedral simulation box, with a minimum distance between AuNP and the box edge set to 1.6 nm. The system was then solvated (TIP3P water model). Sodium chloride was added to neutralize and reach a salt concentration of 150 mM. Fully solvated systems were minimized with the steepest descent method for a maximum of 50,000 steps. All the simulations in this study employed periodic boundary conditions, an integration time step of 2 fs, linear constraints on all bonds involving hydrogen atoms, and a cutoff radius of 1.2 nm for short-ranged nonbonded interactions. The simulations also accounted for long-range electrostatic interactions using the fourth-order particle-mesh Ewald method. All simulations were run in GROMACS v2021.4.^57^

### Steered Molecular Dynamics Simulations

Following energy minimization, the systems were equilibrated and thermalized in the NVT statistical ensemble at a constant rate to a final temperature of 300 K for a total time of 500 ps by using a velocity-rescale thermostat (time constant of 0.1 ps). Two coupling groups of solvent and nonsolvent were used. The systems were then further equilibrated in the NPT ensemble using the Berendsen barostat (time constant 2 ps, reference pressure of 1 bar, and compressibility of 4.5 × 10^–5^ bar^–1^).

Once the systems reached the target temperature and pressure, they were subject to a steered MD simulation in which each analyte was simultaneously pulled away from the AuNP’s center of mass (COM), coupled via a harmonic potential of 2000 kJ mol^–1^ nm^–2^ and a pulling rate of 0.4 nm ns^–1^. This increased rate allowed the unbinding to occur within 10 ns (for ligands 2 nm-long when extended).

During the steered MD simulations, the force acting on the collective variable (CV) was stored every 4 ps and used to reconstruct the PMF profile along the CV. The CV was constructed as the distance between the nanoparticle center of mass and the individual analyte center of masses.

### Umbrella Sampling Molecular Dynamics

Selected configurations from the steered MD simulations of each system were used for the umbrella sampling. Since during steered MD simulations all ten analytes were simultaneously pulled away from the nanoparticle center radially along an independent collective variable, umbrella windows were set up such that each window contained all 10 analytes within the same window using a custom Python code. In this manner, umbrella sampling covers the full range of the collective variable for all analytes without the need to individually sample across each analyte separately. This method provided several advantages. First, it captured a wide range of binding configurations reflecting the structural and dynamic heterogeneities of real systems. Second, the summation of distributions from multiple analytes reduced the noise and improved the statistical uncertainty when compared to averaging separate analyte PMF profiles. A window spacing of 0.2 nm was used in all systems, resulting in a total number of windows between 11 and 17, depending on the nanoparticle shell thickness. Each window was subjected to 2 ns MD simulations (11–17 windows per simulation, each with 10 analytes sampling the monolayers, resulting in a total simulation time of 220–320 ns per system), with configurations restrained using a harmonic potential with a force constant of 1000 kJ mol^–1^ nm^–1^.

The WHAM algorithm, via the *gmx wham* module, was used to reconstruct the free energy profile from the biased simulation. Since each analyte was effectively sampling the same collective variable (the distance radial away from the nanoparticle monolayer) all ten analytes were included in the wham analysis to calculate a single free energy estimate for the unbinding process. To estimate the PMF value of the analyte unbinding for a given system, the unbiased energy difference between the minimum free energy within the monolayer (CV < 3 nm) and the maximum energy outside the monolayer and in solution (CV > 3 nm). Convergence was monitored through the bootstrapping function within the GROMACS WHAM command (Figure S2), along with block analysis (10 blocks) of the standard deviation of the ligand RMSD (Figure S5).

### Hardware, Software, and Performance

All systems underwent simulations on the Franklin HPC cluster at Fondazione Istituto Italiano di Tecnologia. Each simulation utilized one node, which included two AMD EPYC 7713 processors. Each processor uses 60 cores running at 2.0 GHz. 60 MPI processes with 2 OpenMP threads were used per process under a single node. Steered MD was run until the analyte reached the maximum collective variable value, while umbrella sampling was run for a total of 2 ns per window. Overall performance was between 50 and 70 ns/day for umbrella sampling simulations, while steered MD ranged from 80 to 150 ns/day depending on system size.

## Supplementary Material



## Notes

Specifically, linear fits and associated metrics, cluster-wise, for the resulting scoring function (Figure S1), PMF profiles estimated from umbrella sampling for all systems used in this study (Figure S2), all binding free energy estimates from the PMF profiles against experimental free energy values (Figure S3) and detailed RMSD analysis for ligand flexibility alongside block analysis to estimate convergence (Figures S4 and S5); root-mean-square fluctuations of the capping ligands (Figure S6); and average hydrogen bonds formed within the ligand monolayer (Figure S7) (PDF)
