# Supplementary material for: A Scoring Function for Monolayer-Protected Gold Nanoparticles Capable of Recognizing Small Organic Molecules in Solution
Source: J Chem Theory Comput. 2025 Oct 24;21(21):11070–8. doi: 10.1021/acs.jctc.5c01278 (PMC12613317; doi:10.1021/acs.jctc.5c01278)
Supplement: Supplementary file 1 [file ct5c01278_si_001.pdf]

Supporting Information

A Scoring Function for Monolayer-Protected Gold Nanoparticles Capable of Recognizing Small Organic Molecules in Solution

Joseph Wallace,[1] Laura Riccardi,[1] Fabrizio Mancin,[2,*] Marco De Vivo[1,*]

[1] Molecular Modeling and Drug Discovery, Istituto Italiano di Tecnologia, via Morego 30, 16163 Genova, Italy

[2] Department of Chemical Science, University of Padova, Via Marzolo 1, 35131 Padova, Italy

* marco.devivo@iit.it , fabrizio.mancin@unipd.it

Contents

I. Linear Fits to Individual Ligand .... Page 2
Linear Fits to Ligand Clusters for the Ridge Regression-based Scoring Function. (Figure S1)

II. PMF Profile Construction and Distance Distributions .... Pages 3-5
A. Full Umbrella-Sampling PMF Curves for All Systems (Figure S2, Top Panels)
B. Nanoparticle-Analyte Center-of-Mass Distance Distributions Across Umbrella Sampling (Figure S2, Bottom Panels)

II. PMF-Derived vs. Experimental Binding Free Energies .... Page 6
Correlation Plot and Linear Fit Statistics (Figure S3)

III. Ligand Flexibility Analysis (RMSD, block analysis, and RMSF) .... Pages 7-9
Per-Window and Average RMSD for L2 vs. L3 Ligands (Figure S4)
Block analysis of ligand RMSD values. (Figure S5)
RMSF values for all heavy atoms in L2 and L3 ligands. (Figure S6)

IV. Average number of hydrogen bonds formed within the ligand monolayer .... Page 9
Average number of hydrogen bonds formed across all umbrella windows, for both L2 and L3 representative systems (Figure S7)

S1 – Linear fits to ligand clusters for the ridge regression-based scoring function.

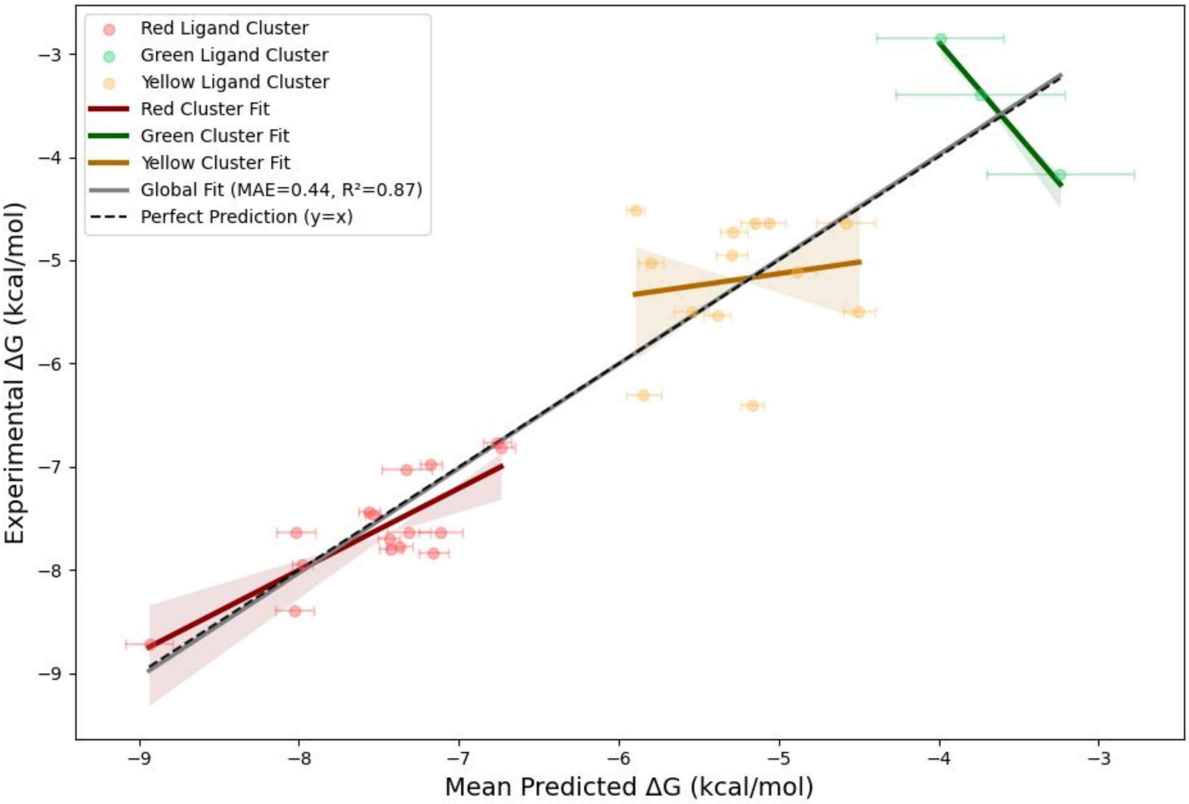

Figure S1 - Ridge-regression predictions of binding free energy (ΔG, kcal/mol) versus experiment using repeated 5-fold cross-validation (1000 repeats) with inner CV for α selection. Points show per-system mean predicted ΔG (x-axis) with ±1 SD as horizontal error bars; colors denote ligand clusters determined from clustering in Figure 1. Solid dark lines are cluster-wise linear fits computed on the per-system means (shaded bands = bootstrap 95% CI). Cluster-wise metrics - Red Cluster: MAE = 0.27, R^2 = 0.59, n = 16; Yellow Cluster: MAE = 0.55, R^2 = -0.32, n = 13, Green Cluster: MAE = 0.80, R^2 = -1.60, n = 13. The gray line is the global linear fit on the per-system means (reported MAE and R^2 in legend), and the black dashed line indicates the identity (y = x).

S2 – Full PMF profiles, from umbrella sampling simulations, for L2- vs L3-AuNPs, each with A10, A18, A19 analytes.

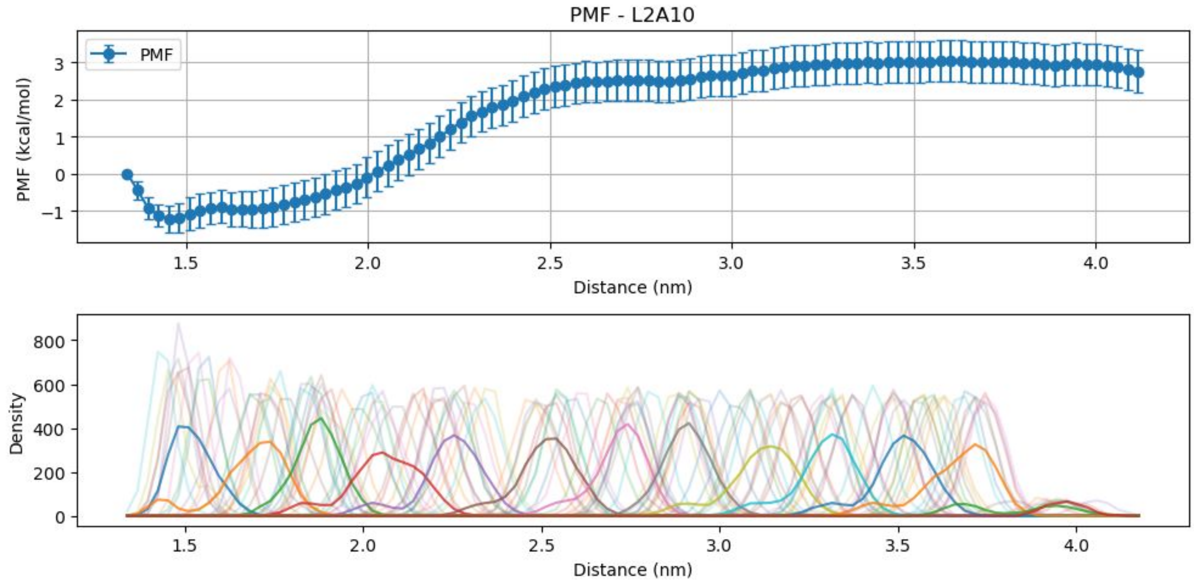

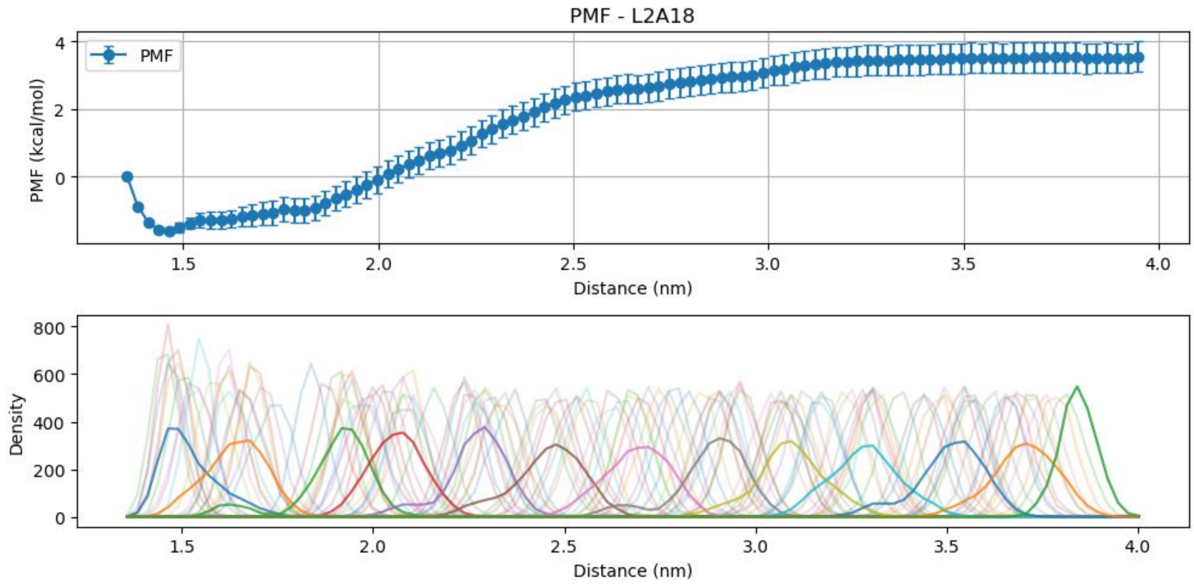

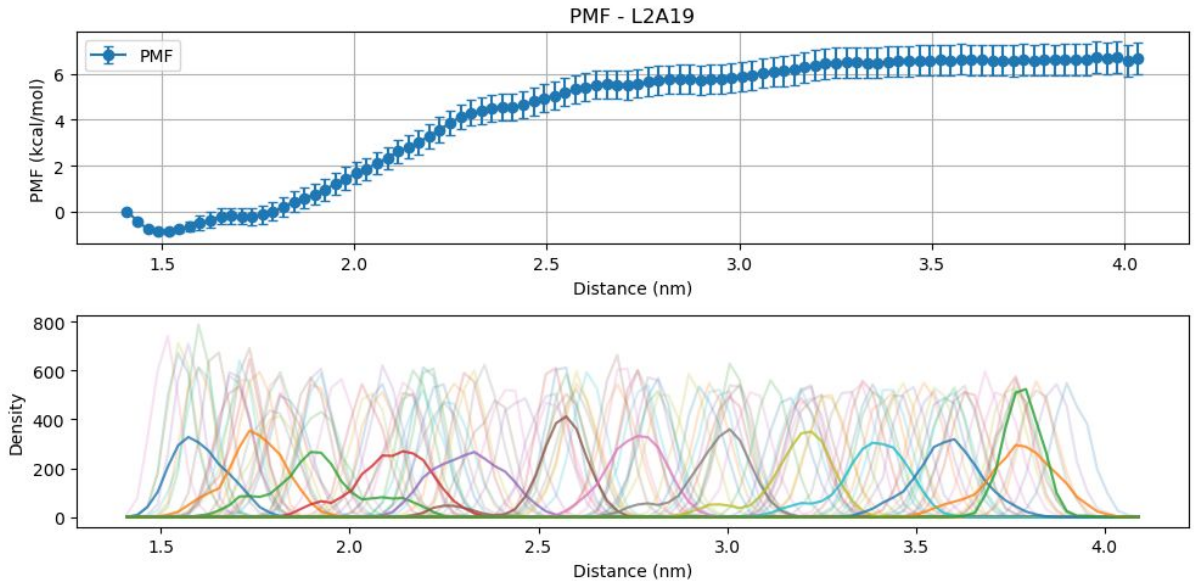
PMF - L2A19
PMF
PMF (kcal/mol)
Distance (nm)
Density
Distance (nm)

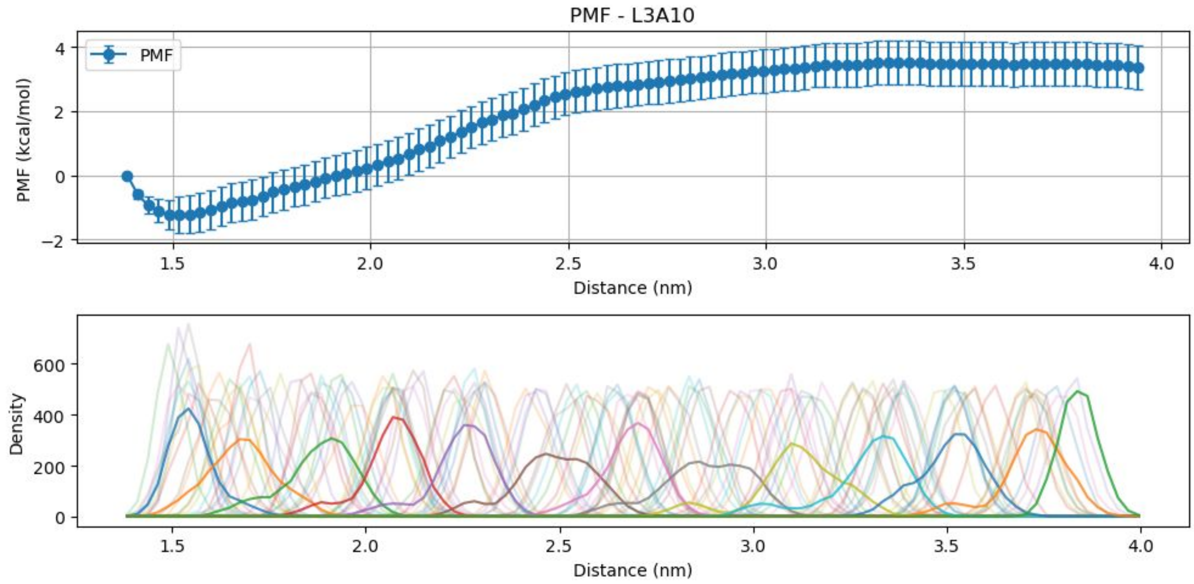
PMF - L3A10
PMF
PMF (kcal/mol)
Distance (nm)
Density
Distance (nm)

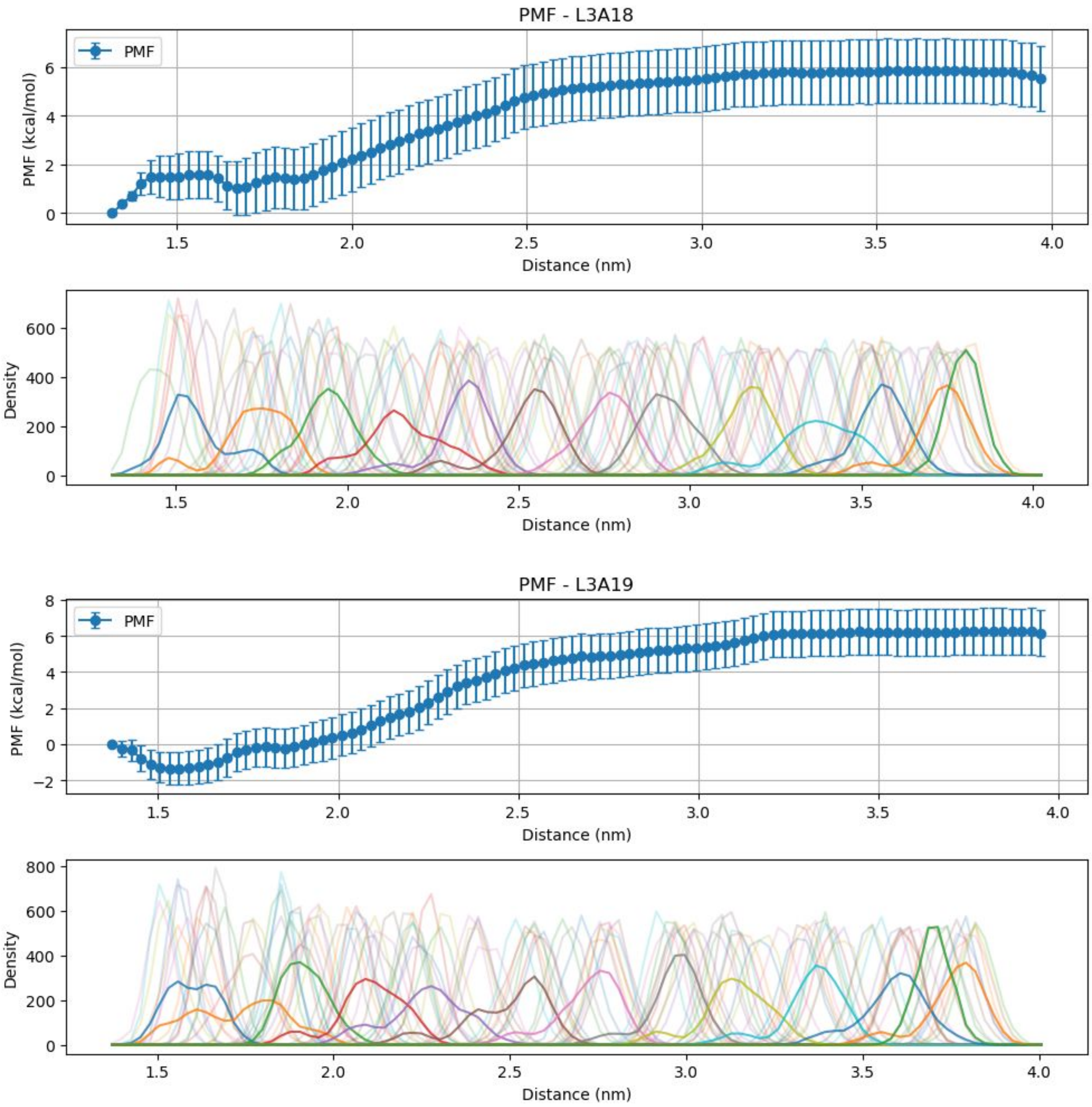

Figure S2. Constructed PMF profiles from umbrella sampling MD simulations for L2- and L3-AuNPs in complex with A10, A18, A19 analytes. Top panels - fully constructed PMF profile. Bottom panels - Distribution of distance between gold nanoparticle centre of mass and analyte centre of mass for all windows. Bold lines are averages over each simulation, taken from the combined distribution from all analytes (translucent lines).

S3 – Binding free energy estimates, from umbrella sampling simulations *vs* experimental binding free energy for AuNPs capped with L2 or L3 in complex with A10, A18, A19.

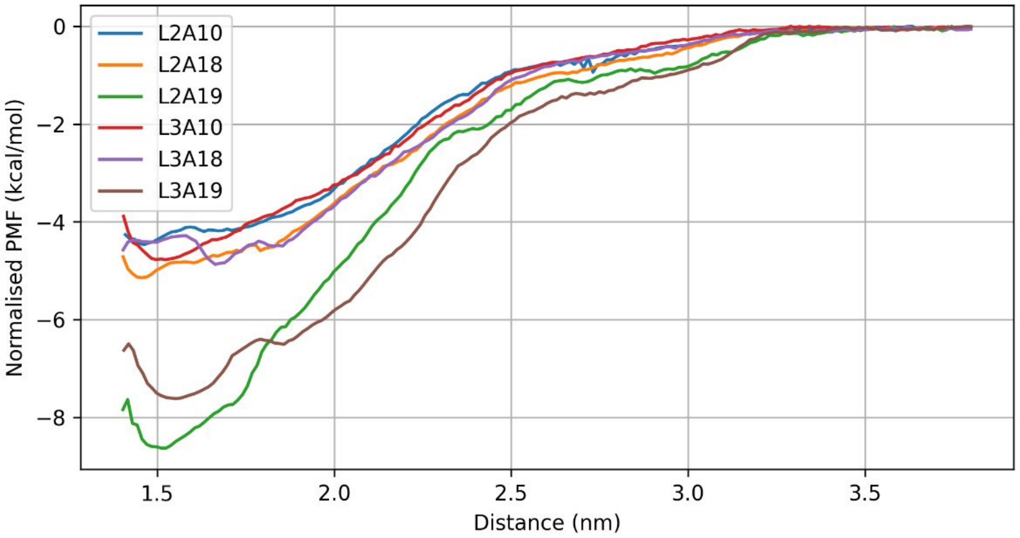

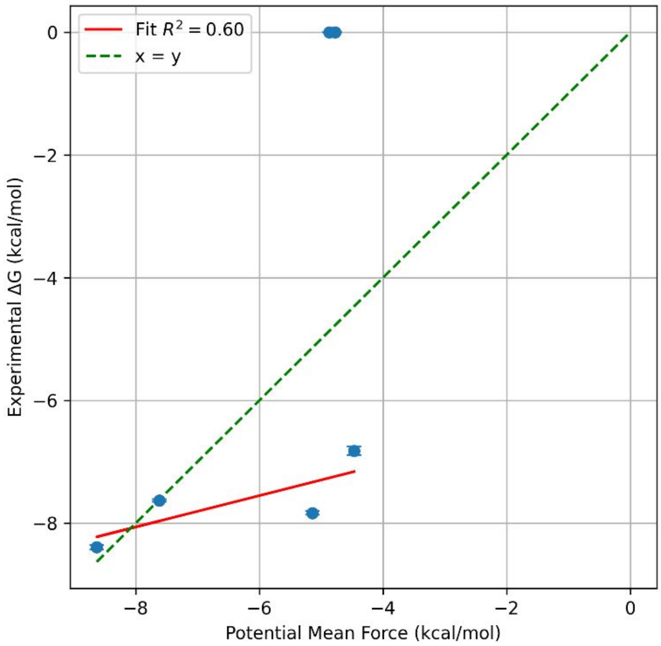

Figure S3. Potential mean force estimates from umbrella sampling molecular dynamics for L2- and L3-AuNP in complex with A10, A18 and A19 analytes. Top: Average Potential Mean Force profile across the full range of distance between AuNP and analyte centre-of-mass. Bottom: Computed free energy difference (kcal/mol) between bound and unbound states against experimental binding free energy (kcal/mol). Blue circles represent individual AuNP–analyte systems, alongside experimental errors, the dashed green line denotes perfect agreement (y = x), and the solid red line is the least-squares fit (R^2 = 0.60).

S4 – Root mean square deviation of ligand atom positions.

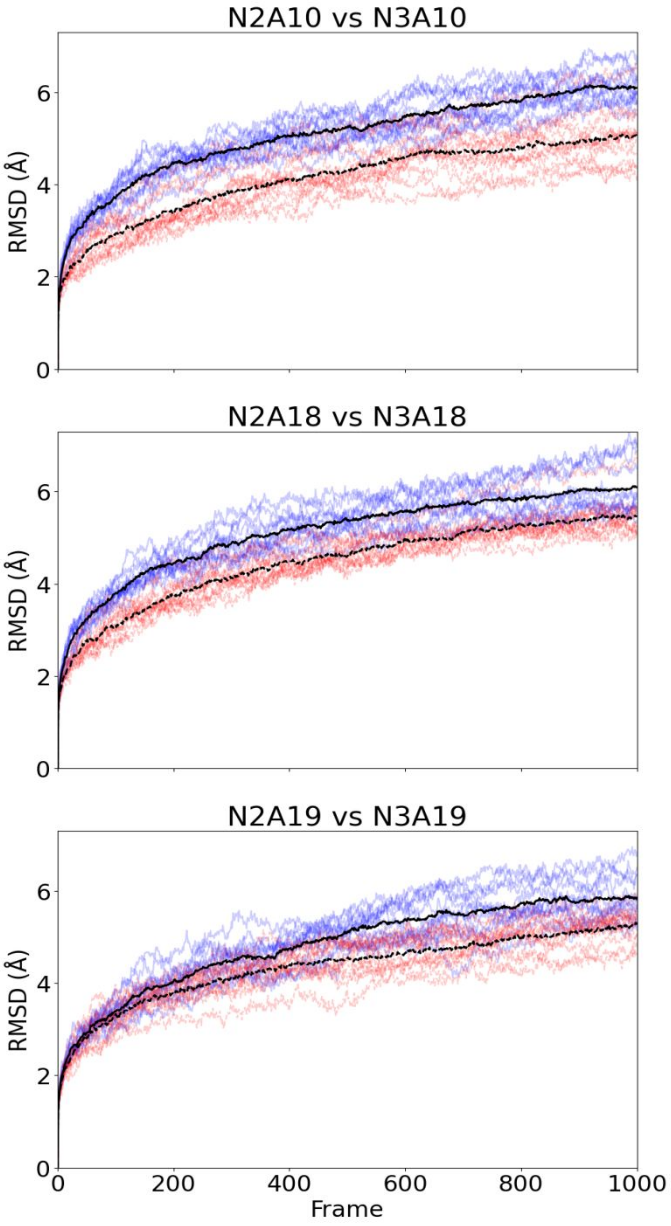

Figure S4. RMSD across all simulation widows (Red: L3, Blue: L2). Averaged RMSD over all windows are shown in black (Solid line: L3, Dashed line: L2).

S5 – Block analysis of root mean square deviation of ligand atom positions.

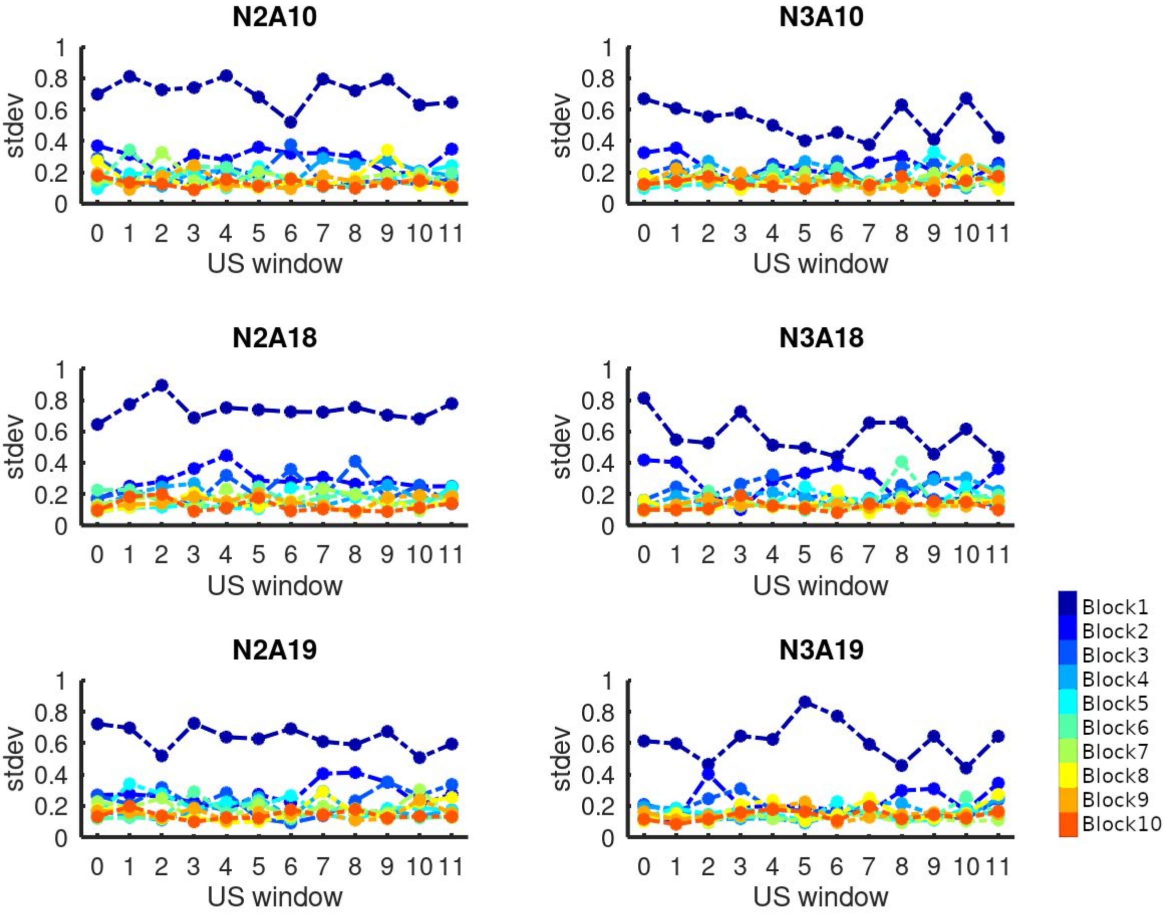

Figure S5. Block analysis of RMSD standard deviation aross all umbrella sampling windows, for the six representative AuNP-analyte systems. Each line corresponds to one of ten equal trajectory blocks (colour coded – see legend), showing the variablity of RMSD.

S6 – Root mean square fluctuations of all heavy atoms for L2 and L3 systems.

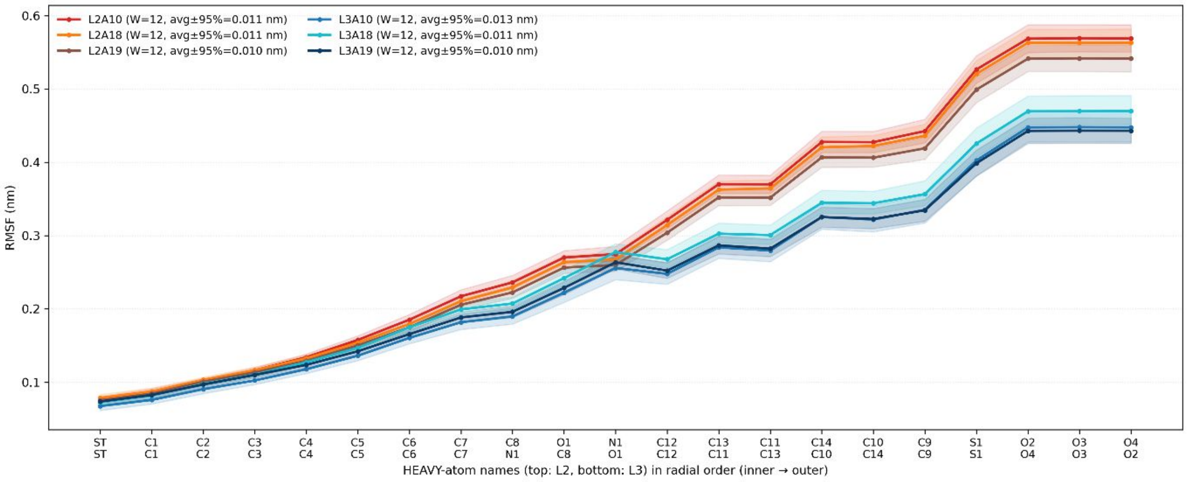

Figure S6. Root mean square fluctuations (RMSF of ligand heavy atoms for L2- and L3-capped AuNP complexes with analytes A10, A18, and A19. Atom labels are ordered radially from the nanoparticle attachment point outward. Shaded regions represent 95% confidence intervals estimated from block averaging (12 windows). L2 ligands consistently show enhanced fluctuations relative to L3, consistent with greater conformational flexibility. As expected, RMSF values increase along the chain length, moving outward from the nanoparticle core.

S7 – Average number of hydrogen bonds formed across all umbrella windows, for both L2 and L3 representative systems.

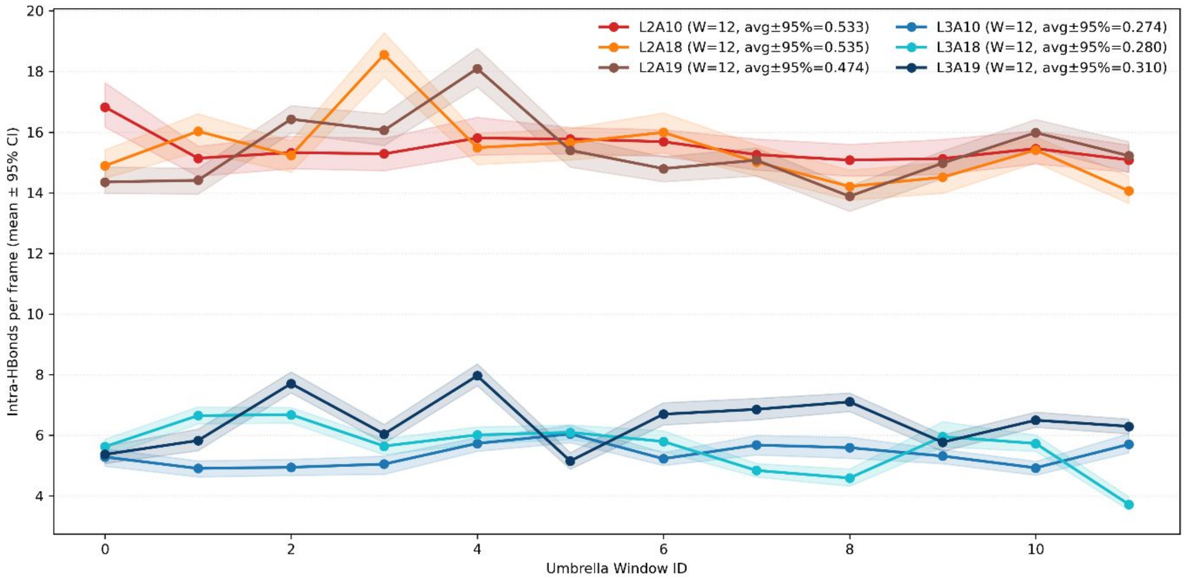

Figure S7. Average number of inter-ligand hydrogen bonds per frame across umbrella sampling windows for L2- and L3-capped AuNP complexes with analytes A10, A18, and A19. Shaded regions represent 95% confidence intervals estimated from block averaging (12 windows). L2 systems consistently formed more inter-ligand hydrogen bonds than L3, suggesting that these transient interactions support a cooperative, dynamic, and adaptable monolayer environment.
